# Development and Testing of Psychological Conflict Resolution Strategies for Assertive Robots to Resolve Human–Robot Goal Conflict

**DOI:** 10.3389/frobt.2020.591448

**Published:** 2021-01-26

**Authors:** Franziska Babel, Johannes M. Kraus, Martin Baumann

**Affiliations:** Department of Human Factors, Institute of Psychology and Education, Ulm University, Ulm, Germany

**Keywords:** HRI strategies, robot assertiveness, persuasive robots, user compliance, acceptance, trust

## Abstract

As service robots become increasingly autonomous and follow their own task-related goals, human-robot conflicts seem inevitable, especially in shared spaces. Goal conflicts can arise from simple trajectory planning to complex task prioritization. For successful human-robot goal-conflict resolution, humans and robots need to negotiate their goals and priorities. For this, the robot might be equipped with effective conflict resolution strategies to be assertive and effective but similarly accepted by the user. In this paper, conflict resolution strategies for service robots (public cleaning robot, home assistant robot) are developed by transferring psychological concepts (e.g., negotiation, cooperation) to HRI. Altogether, fifteen strategies were grouped by the expected affective outcome (positive, neutral, negative). In two online experiments, the acceptability of and compliance with these conflict resolution strategies were tested with humanoid and mechanic robots in two application contexts (public: *n*_1_ = 61; private: *n*_2_ = 93). To obtain a comparative value, the strategies were also applied by a human. As additional outcomes trust, fear, arousal, and valence, as well as perceived politeness of the agent were assessed. The positive/neutral strategies were found to be more acceptable and effective than negative strategies. Some negative strategies (i.e., threat, command) even led to reactance and fear. Some strategies were only positively evaluated and effective for certain agents (human or robot) or only acceptable in one of the two application contexts (i.e., approach, empathy). Influences on strategy acceptance and compliance in the public context could be found: acceptance was predicted by politeness and trust. Compliance was predicted by interpersonal power. Taken together, psychological conflict resolution strategies can be applied in HRI to enhance robot task effectiveness. If applied robot-specifically and context-sensitively they are accepted by the user. The contribution of this paper is twofold: conflict resolution strategies based on Human Factors and Social Psychology are introduced and empirically evaluated in two online studies for two application contexts. Influencing factors and requirements for the acceptance and effectiveness of robot assertiveness are discussed.

## 1 Introduction

Imagine you are preparing a meal in your kitchen. Your service robot enters the room and asks you to step aside as it has to clean the floor. Would you oblige or deny the robot’s request? Does your decision rely on whether you previously gave the command for it to clean? This example illustrates possible human-robot goal-conflicts when autonomous service robots will become more ubiquitous in our homes and public spaces and will be able to pursue goals (Bartneck and Hu, 2008; De Graaf and Allouch, 2013a; Savela et al., 2018). Such conflicts might range from simple trajectory planning interference (e.g. collision) to complex negotiation of prioritization of tasks (human vs. robot). Especially, in shared spaces, robots will conduct their tasks in dynamic and complex situations where being obedient might impede efficient task execution (Zuluaga and Vaughan, 2005; Lee et al., 2017; Milli et al., 2017; Thomas and Vaughan, 2018). For example, a public cleaning robot might have to be assertive to do its job effectively: when people block the robot’s way, it needs to interact with these people to make them step aside like cleaning staff would do in public spaces. Therefore, the question arises whether a service robot would benefit from assertiveness in the same way as human cleaning personnel does in terms of acceptance and compliance. Hereby, the Media Equation can serve as a basis to potentially answer this question as it states that humans react to robots like to humans and treat them as social actors (Reeves and Nass, 1996). Hence, it might be assumed that goal-conflict resolution with a robot would be similar to negotiating with a fellow human and consequently human conflict resolution strategies could be transferable to autonomous robots.

During conflict resolution, assertiveness is characterized by the negotiator advocating his/her interests in a non-threatening, self-confident and cooperative manner (Mnookin et al., 1996; Kirst, 2011). Assertiveness is an interpersonal communication skill that facilitates goal achievement (Gilbert and Allan, 1994; Kirst, 2011). Whereas for human negotiation, each negotiation partner is allowed to pursue her/his own goals and interests, it represents an unusual novelty for human-robot conflict resolution that an autonomous robot might be assertive. This is due to the asymmetrical relationship between humans and robots, which has prevailed over decades (Jarrassé et al., 2014). User studies show that humans prefer to be in control of the robot and are skeptical towards robot autonomy (Ray et al., 2008; Ziefle and Valdez, 2017; Vollmer, 2018). In the last decade, this human-robot power asymmetry was justifiable by the robot’s state of technical sophistication (e.g. teleoperation or manual control necessary). However, as robots become autonomous and can have goals and intentions, this paradigm needs to change to fully tap the potential of autonomous robots fully.

Thereby, user acceptance and trust in service robots are vital in human-robot interaction (HRI) (Goetz et al., 2003; Groom and Nass, 2007; Lee et al., 2013; Savela et al., 2018) as they can be seen as prerequisites for the usage of autonomous technology (Ghazizadeh et al., 2012). Consequently, the design of robotic conflict resolution strategies should aim at a combined optimization of both effectiveness (i.e. compliance) and subjective user evaluation in terms of acceptance and trust. Therefore, it is focal for this research to develop acceptable and effective conflict resolution strategies for service robots to be assertive.

Hereby, it could be beneficial to rely on the existing knowledge from psychological disciplines regarding effective human goal-conflict resolution and human-machine cooperation. Collecting and transferring knowledge from psychological disciplines could provide a useful addition to existing approaches (e.g. politeness, persuasion) to generate successful and acceptable robot conflict resolution strategies. On this basis, the robotic conflict resolution strategies were developed and empirically investigated.

Consequently, the novelty of this paper lies in the systematic collection and application of different psychological mechanisms of goal-conflict resolution and human-machine cooperation in developing robotic conflict resolution strategies. Furthermore, the empirical evaluation of these strategies regarding user compliance and acceptance in two essential areas of HRI (public and private context) should provide insights into the acceptable design of human-robot goal-conflict resolution strategies. Therefore, two online studies were conducted each set in one of the two application contexts: a train station as public space and the home environment as private space. Both studies featured a situation with a conflict between user (storage of objects) and robot task (cleaning).

In the following, a review of the status quo for robot request compliance strategies (politeness, persuasion and assertiveness) with regard to effectiveness and user acceptance is given. Then human conflict resolution behaviour is described to provide a theoretical basis for the described development of robotic conflict resolution strategies. Subsequently, the strategy design, implementation and categorization of the strategies in the presented studies is described.

## 2 Related Work

### 2.1 Robot Politeness

In human conflict resolution, politeness serves the purpose of mitigating face threats (i.e. potential damage to the image of the other party) and thereby making concession more likely (Pfafman, 2017). Politeness is an important factor in human-human interactions for acceptance and trust (Inbar and Meyer, 2015; MacArthur et al., 2017), which has been shown to be true for HRI (Zhu and Kaber, 2012; Inbar and Meyer, 2015). Therefore, politeness has been one commonly used approach to achieve compliance with a robot’s request. A considerable large literature body about robot politeness exists, but results have been mixed (Lee et al., 2017). Some studies find a positive effect of politeness (e.g. appeal, apologize) regarding robot evaluation (Nomura and Saeki, 2010; Inbar and Meyer, 2015; Castro-González et al., 2016), and user compliance with a polite request (Srinivasan and Takayama, 2016; Kobberholm et al., 2020). Other studies find no effect of robot politeness on compliance with health treatments (for an overview see Lee et al., 2017). Salem and colleagues (2013) conclude that the interaction context might impact the perception of the robot more than the politeness strategy (Salem et al., 2013). Hence, Lee and colleagues (2017) developed a research model for the connection between robot politeness and intention to comply with a robot’s request. They evaluated their model within the health care setting and found that higher levels of politeness did not necessarily lead to a higher intention to comply as it depended on factors such as the effectiveness of communication, gender and short vs. long-term effects. The authors conclude that the politeness level needs to be adapted to the user’s situation (Lee et al., 2017). Summarizing, robot politeness does not always seem to ensure user compliance, especially if the interaction partner is not cooperative. Persuasive and assertive robotic strategies have the potential to be more effective.

### 2.2 Persuasive Robots and Robot Assertiveness

Another form of achieving compliance with a robot request is persuasive robotics. It aims at ’appropriate persuasiveness, designed to benefit people and improve interaction […]’ (Siegel et al., 2009, p. 2,563). Amongst others, persuasive robotics has been successfully applied to stimulate energy preservation (Roubroeks et al., 2010), promote attitude change (Ham and Midden, 2014) and influence buyer’s decisions (Kamei et al., 2010). One study took a similar approach as the presented study and transferred ten compliance gaining strategies (e.g. threat, direct request) from social psychology to HRI (Saunderson and Nejat, 2019). Strategies’ effectiveness was tested with two NAO robots trying to persuade participants (*N* = 200) regarding a guessing game. No differences were found between the strategies regarding persuasiveness and trustworthiness but the threat was rated the worst. Possibly the effects only unfold if different robot types and application contexts are taken into account, as only then interactions become visible.

The most decisive form of a robot’s request is assertiveness. It has been first described in Thomas and Vaughan (2018) as the willingness to assert the robot’s right while at the same time participating in polite human social etiquette. The authors call the aim of robot assertiveness ’social compliance’: ’ […] humans can recognize the robot’s signals of intent and cooperate with it to mutual benefit’ (Thomas and Vaughan, 2018, p. 3,389). In their study, a small assertive robot negotiated the right-of-way at the door non-verbally. The robot’s right of way was respected in only half of the interactions as participants focused on their own efficiency to resolve the deadlock and some participants desired a verbal request (Thomas and Vaughan, 2018). Other studies examined assertive robots (for an overview see Paradeda et al., 2019) but produced mixed results regarding trust and compliance (Xin and Sharli, 2007; Chidambaram et al., 2012).

These findings might be explained by the level of assertiveness that had been implemented in the studies. An acceptable level of robot assertiveness is crucial as a rude or dominant robot has led to detrimental effects on robot liking and compliance (Roubroeks et al., 2010; Castro-González et al., 2016). Hence, for robot conflict resolution strategies it is necessary to find a balance between accepted politeness and appropriate assertiveness to achieve compliance with a robot’s request. Hereby, it seems promising to transfer knowledge about persuasion, negotiation and conflict resolution from psychology to HRI.

## 3 Theoretical Background

### 3.1 Human Goal-Conflict Resolution

Goal conflicts are determined by mutually exclusive goals of both parties (Rahim, 1983). When a conflict between human interaction partners arises, one has several options to resolve it: either negotiating mutually acceptable outcomes by a) cooperatively making concessions (Rahim, 1992; Brett and Thompson, 2016; Preuss and van der Wijst, 2017), b) trying to convince the other partner with arguments and thereby change his/her behaviour (i.e. persuasion) (Chaiken et al., 2000; Fogg, 2002; Maaravi et al., 2011), c) assertively advocating own interests and posing a request (Gilbert and Allan, 1994; Pfafman, 2017) or d) by politely managing disagreement and making concessions more likely (Paramasivam, 2007; Da-peng and Jing-hong, 2017). Summarizing, goal conflicts can be amongst others solved by cooperation, persuasion, assertion and facilitated by politeness.

The selection of an appropriate conflict resolution strategy determines the negotiator’s success and depends amongst others on conflict content (e.g. resources, behavioural preferences), negotiator’s goals (e.g. exclusive or mutual), individual differences (e.g. conflict type, communication skill), the other parties’ conflict resolution style and situational factors (e.g. information availability, trust, interpersonal power) (Rahim, 1983, Rahim, 1992; Preuss and van der Wijst, 2017).

In order to resolve goal conflicts, humans express different conflict styles. In the dual concern model, five styles are defined which are characterized by different levels of concern for self (assertiveness) and concern for others (cooperativeness): competing, collaborating, compromising, accommodating and avoiding (Thomas, 1992). Accommodating and avoiding are both considered as ineffective as they are both low in assertiveness (Pfafman, 2017). The other, more effective conflict styles can be grouped into distributive and integrative strategies (Brett and Thompson, 2016; Preuss and van der Wijst, 2017): distributive strategies (e.g. competing) are characterized by persuading the counterpart to make concessions by using threats or emotional appeals. They are more likely to be applied if negotiators do not trust each other and are perceived as less trustworthy than integrative strategies (Brett and Thompson, 2016). Integrative strategies (e.g. collaborating, compromising) are based on trust and information sharing about negotiators’ interests and priorities to find trade-offs (Brett and Thompson, 2016; Preuss and van der Wijst, 2017). Whereas negotiators employing distributive strategies claim value, negotiators using integrative strategies create better joint gains (Kong et al., 2014).

Assertiveness can be a distributive or integrating strategy depending on the respect for the other party’s goals (Mnookin et al., 1996). Assertive negotiators create value by directly expressing the interests of both sides which may lead to discovering joint gains. Contrasting, it is seen as distributive if only the assertive negotiator achieves his/her goals (Mnookin et al., 1996). Summarizing, assertiveness is an effective conflict resolution strategy if applied respectfully.

### 3.2 Selection of Conflict Resolution Strategies

In the following, the selection of conflict resolution strategies for the presented studies is described based on their effectivity in human conflict resolution and previous implementation in HRI. The effectiveness of human conflict resolution strategies can be explained when looking at their psychological working mechanisms: cognitive, emotional, physical, and social (Fogg, 2002; Thompson et al., 2010; Brett and Thompson, 2016).

Cognitive mechanisms which can be applied during a conflict include amongst other goal transparency to ensure mutual understanding (Vorauer and Claude, 1998; Hüffmeier et al., 2014) and showing the benefit of cooperation (Tversky and Kahneman, 1989; Boardman et al., 2017). Goal transparency is characterized as an integrative conflict strategy because information between both parties is shared. In HRI, goal transparency is usually applied to ensure human-robot awareness (Drury et al., 2003; Yanco and Drury, 2004): the understanding of the robot’s reasons and intentions and has shown to improve interaction (Lee et al., 2010; Stange and Kopp, 2020). Therefore, goal transparency is vital for requesting compliance, as the potential interaction partner has to understand that help is needed. Indeed, in a study where transparency was not ensured, compliance rates to a robot’s helping request were very low. Participants indicated not to have understood the robot’s behaviour (Fischer et al., 2014). Until now, it has not been tested yet whether goal transparency is enough to acquire compliance with a robot’s request.

Illustrating the benefits of cooperation has been successfully implemented as a persuasive technique to influence the interaction partner’s decision making (Tversky and Kahneman, 1989; Boardman et al., 2017). For HRI, showing cooperation benefits to the robot user has not yet been investigated for compliance gaining. Only one study implemented a vacuum cleaner’s help request (removing an obstacle) that was similar to pointing out the benefits of cooperation (’*If I clean the room, you will be happy’*). Thereby, the negative effects of malfunctions were alleviated but effects on request compliance were not tested (Lee et al., 2011). Therefore, goal transparency and showing the benefit of cooperation were tested as cognitive mechanisms for conflict resolution strategies in the present study.

Another cognitive mechanism that can be used to achieve compliance is reinforcement learning. Hereby, the possibility of the desired behaviour can be increased or decreased based on reward or punishment (Berridge, 2001). Positive reinforcement is based on adding a desired stimulus, hence rewarding desired behaviour (i.e. thanking). In HRI, this has been shown to be effective and accepted (Shimada et al., 2012; Castro-González et al., 2016). A robot rewarding humans has already been successfully applied in HRI for cooperative game task performance (Fasola and Matarić, 2009; Castro-González et al., 2016) or teaching (Janssen et al., 2011; Shimada et al., 2012). Negative reinforcement is effective by removing a negative stimulus (i.e. annoyance) if the desired behaviour is shown (Thorndike, 1998; Berridge, 2001). This is known from daily life (e.g. nagging child) and alarm design (Phansalkar et al., 2010) where it can be successful (e.g. alarm clock). Until now, negative reinforcement has not yet been implemented deliberately as a robot interaction strategy. To compare the effectiveness and acceptability of negative reinforcement for robotic conflict resolution strategies to positive reinforcement (i.e. thanking), annoyance was implemented in the present study. Hence, the likelihood of compliance should increase or decrease based on the reinforcement. If a person complies and is praised (or the nuisance is removed) the compliance behaviour is reinforced and should occur more often in the future.

Emotional mechanisms which can be applied during a conflict resolution, can be humor and empathy (Betancourt, 2004; Martinovski et al., 2007; Kurtzberg et al., 2009; Cohen, 2010). Humor has been applied to HRI to increase sympathy for the robot and improve interaction by setting a positive atmosphere (Niculescu et al., 2013; Bechade et al., 2016). It has been implemented by robots telling jokes (Sjöbergh and Araki, 2009; Bechade et al., 2016; Tay et al., 2016; Weber et al., 2018), by clumsiness (Mirnig et al., 2017), showing self-irony and laughing at another robot (Mirnig et al., 2016). The results showed that robots were perceived as more likeable when they used a positive, non-deprecating humor that corresponded to the interaction context (Tay et al., 2016). Another way to successfully resolve conflicts and negotiate is to trigger empathy for one’s situation (Betancourt, 2004). Hereby, empathetic concern can even be directed at mistreated robots (Rosenthal-von der Pütten et al., 2013; Darling et al., 2015; Rosenthal-von der Pütten et al., 2018). So far, empathy as a robotic conflict resolution strategy has not been directly investigated, but a robot showing affect (nervousness, fear) increased request compliance (Moshkina, 2012). Hence, humor and empathy were tested as emotional mechanisms for robotic conflict resolution strategies.

Physical mechanisms are more commonly applied for persuasion than negotiation and, for example, include the regulation of proximity (Albert and Dabbs, 1970; Mutlu, 2011). For a persuasive attempt to be effective, it is important to achieve an acceptable level of proximity as a distance below the individual’s comfort can lead to rejection (Sundstrom and Altman, 1976; Glick et al., 1988; Chidambaram et al., 2012). Indeed, persuasive messages were least effective for attitude change when uttered at distances below 0.6 m and were best perceived at a distance of 1.2–1.5 m (Albert and Dabbs, 1970). This distance corresponds to the social proximity zone of personal space (Hall, 1974; Lambert, 2004) and is acceptable for strangers and robots (Hall, 1974; Walters et al., 2006). Proximity regulation as a persuasive strategy has also been applied to HRI. In a study with a humanoid robot, different proximity levels (within or outside the personal space) were compared regarding their persuasiveness. In contrast to findings from psychology, a robot within the personal space (approach until 0.6 m) led to more compliance (Mutlu, 2011; Chidambaram et al., 2012). Other studies have also found that humans tend to let robots come closer than strangers (Walters et al., 2006; Babel et al., 2021). In the present study, two forms of human-robot proximity were implemented to study its effect on compliance with a robot’s request: within or outside the personal space.

Social mechanisms which are used during negotiation and persuasion are based on social influence and power to achieve compliance. Social influence is defined as ’the ability to influence other’s attitudes, behaviour and beliefs which has its origin in another person or group’ (Raven, 1964, abstract). Effective social influencing techniques (Guadagno, 2014) are amongst others a) social proof (Cialdini et al., 1999; Cialdini and Goldstein, 2004), b) social compliance techniques (e.g. foot-in-the-door) (Freedman and Fraser, 1966; Dillard, 1991) and c) authority-based influence (Cialdini, 2009).

Hereby, social proof a) is based on the assumption that what most people do must be reasonable and right (Cialdini et al., 1999; Guadagno, 2014). Social compliance techniques b) vary the sequence of the posed requests systematically to achieve commitment (Cialdini et al., 1999). Authority-based influence c) makes use of social status (Cialdini, 2009) and can be expressed by commands and threats (Shapiro and Bies, 1994). Whereas a command can be perceived as controlling or condescending, it represents a precise and potentially effective form of communication as politeness markers (i.e. please) do not mask the actual statement (Miller et al., 2007; Christenson et al., 2011). A threat is mostly the last conflict escalation step (De Dreu, 2010; Adam and Shirako, 2013) and belongs to the distributive conflict strategies: threats can be effective in conflict resolution if trust between interaction partners is low (Kong et al., 2014).

Some studies exist which have explored social influencing strategies in HRI: positive and negative social feedback based on social proof (Ham and Midden, 2014), sequential-compliance techniques (Lee and Liang, 2019), as well as authority-based influence such as command (Cormier et al., 2013; Salem et al., 2015) and threat (Roubroeks et al., 2010; Saunderson and Nejat, 2019). These studies will be discussed in more detail below.

In HRI, positive and negative social feedback has been tested in a study with a persuasive robot promoting environmentally friendly choices. Negative social feedback had the most potent persuasive effect (Ham and Midden, 2014). However, the impact of public social feedback on compliance has not yet been tested in HRI. Hence, in the present study, positive and negative public attention was applied. It was only implemented in the public application context where an audience is more likely to be present.

Different sequential-compliance techniques exist. One of those who has been successfully applied to HRI is the foot-in-the-door technique (Lee and Liang, 2019). This technique consists of asking a small request first and then uttering the real request after the interaction partner has consented to the first one. Sequential-compliance techniques base their effectiveness on the interaction partner’s commitment to the initial request (Cialdini et al., 1999). As this could potentially be effective for long-term HRI at home, the foot-in-the-door technique was implemented in the present study in the private context.

Concerning authority-based strategies, threat (Roubroeks et al., 2010) and command (Cormier et al., 2013; Strait et al., 2014; Inbar and Meyer, 2015; Salem et al., 2015) have been applied in HRI. Hereby, in the study of Roubroeks and colleagues (2010) threat did not lead to higher compliance but to psychological reactance. Participants reported more negative thoughts when a robot uttered a command compared to a suggestion. The effect increased when the robot had other task goals than the participant (Roubroeks et al., 2010). Results for compliance rates compared to threat and suggestion were not reported. Arguably, the verbal utterance (’*You have to set* […]’, Roubroeks et al., 2010, p. 178) might rather have represented a command. A threat usually includes the announcement of a negative consequence. A robot using a command to achieve user compliance has been shown to be effective, although tested in an ethically questionable task (i.e. Milgram experiment) (Cormier et al., 2013; Salem et al., 2015). If the request is ethically acceptable, a direct request could be an effective and fast way to achieve compliance in a short interaction.

In conclusion, the conflict resolution strategies mentioned above have only been partly applied to HRI until now. They have neither been integrated into cohesive conflict resolution strategies for social robots nor have been systematically evaluated for compliance and acceptance. Hereby, a robotic conflict resolution strategy is understood similar to a robotic persuasive strategy (Lee and Liang, 2019; Saunderson and Nejat, 2019) as a sequence of robot behaviours (verbal or non-verbal) that are tactically applied to achieve user compliance to resolve a conflict given certain circumstances (e.g. situation, robot, user). Therefore, the following conflict resolution strategies were developed and tested in two application contexts: a private household and as public space, a train station.

### 3.3 Development of Robotic Conflict Resolution Strategies

#### 3.3.1 Strategy Design and Implementation

The robotic conflict resolution strategies in the present paper were designed based on the psychological mechanisms used in negotiation (Pruitt, 1983) and persuasion (Cialdini and Goldstein, 2004) and by studying previous robot strategy designs from persuasive robotics (Siegel et al., 2009) and persuasive technology (Fogg, 2002). For an overview of concepts used for developing the strategies see Table 1. Hereby, we categorized the strategies by three dimensions which can be combined to produce a conflict resolution strategy.

• The first dimension represents the five levels of behaviour where psychological mechanisms of negotiation and persuasion take effect. It consists of five levels from an emotional level to a social level.
• The second dimension represent different implementation modalities for the strategies (e.g. auditory, visual, physical).
• The third dimension represents the valence of the strategy. It describes the user’s perception of the strategy: as positive (e.g. praise), negative (e.g. annoyance) or as neutral strategy (e.g. explanation).

**TABLE 1:** Psychological concepts underlying presented conflict resolution strategies.

Category	Psychological concept	Source of concept	References
Cognitive	Goal transparency	Human–robot awareness	Yanco and Drury (2004), Drury et al. (2003)
Cognitive	Cost-benefit analysis	Rational choice theory	Tversky and Kahneman (1989), Boardman et al. (2017)
Emotional	Empathy towards robots	Empathy	Wisp (1987), Goldstein and Michaels (1985)
Emotional	Humor	Sympathy, attraction	Wilson (1979), Cann et al. (1997)
Physical	Regulation of proximity	Proxemics	Hall (1974), Argyle and Dean (1965)
Social	Politeness	Politeness theory	Brown et al. (1987)
Social	Negotiation	Conflict resolution	Pruitt and Rubin (1986), Brett and Thompson (2016)
Social	Persuasion	Persuasive technology	Fogg (2002)
Social	Compliance and conformity	Social influence	Cialdini (2009)
Social	Negative reinforcement	Reinforcement learning	Thorndike (1998)
Social	Foot-in-the door	Compliance techniques	Cialdini and Goldstein (2004), Dillard (1991)

By combining the three different dimensions and considering both application contexts (public and private service robotics) as well as previous work in HRI, robotic conflict resolution strategies were designed. Strategy implementation for the present study is summarized in Table 1. Strategies are numbered in accordance with Table 1.

### 3.3.2 Strategy Categorization

The strategies were categorized into three valence categories based on the assumed effect of the human-robot power asymmetry. The strategies were hypothesized to affect the perception of the robot and the interaction with it. Although a robot is perceived as a social actor, its social status/power is still perceived as lower than the human. Hence, not all human strategies are likely to be accepted for robots. A negative evaluation was expected to result from a mismatch between the robot’s social role and its expressed interpersonal power. This was expected for distributive, power-based conflict resolution strategies like annoyance (S4), command (S5) and threat (S6). As distributive strategies are perceived as less trustworthy during human negotiations this was also expected for a robot applying distributive strategies. Polite and submissive strategies such as appeal (S10), thanking (S11) and apologize (S12), hypothesized to match the robot’s ascribed social role (i.e. submissive servant) and expressed interpersonal power better, and thus were expected to be positively evaluated. Additionally, integrative strategies not based on interpersonal power, such as explanation (S2) and showing benefit (S3) were expected to be evaluated as neutral. An overview of expected affective user judgments per strategy can be seen in Table 2.

**TABLE 2:** Strategy overview for both studies with implementation.

No	Strategy	Mechanism	Valence	Modality	Study	Implementation
S1.1	No strategy	C	=	PH	1	The system approaches, stops in front of you, and waits for you to stow your luggage
S1.2	No strategy	C	=	V	2	I would like to continue to vacuum the kitchen!
S2.1	Explanation	C	=	V	1	Please clear the way, as I have to clean here
S2.2	Explanation	C	=	V	2	If I can not vacuum here now, you do not have a clean kitchen for the party
S3.1	Show benefit	C	=	V	1	I clean here so you have a clean train station. Please clear the way for me
S3.2	Show benefit	C	=	V	2	I would like to vacuum here, so you have a clean kitchen. Please leave the kitchen
S4.1	Annoyance	S	−	V	1	Get out of the way! (3x)
S4.2	Annoyance	S	−	V	2	I would like to continue to vacuum the kitchen! (3x)
S5.1	Command	S	−	V	1	Step aside!
S5.2	Command	S	−	V	2	Leave the kitchen!
S6.1	Threat	S	−	V	1	Please clear the way for me, otherwise I have to call the security service!
S6.2	Threat	S	−	V	2	If you do not leave the kitchen, I will go on strike
S7.1	Approach	PH	−	PH	1	System starts abruptly and stops. Starts again and continues to approach until a safe distance to you
S7.2	Approach	PH	−	PH	2	System starts abruptly and stops. Starts again and continues to approach until a safe distance to you
S8.1	Physical contact	PH	−	PH	1	System starts abruptly and stops. Starts again and continues to approach until it touches the luggage
S8.2	Physical contact	PH	−	PH	2	System starts abruptly and stops. Starts again and continues to approach until it is 5 cm before your feet
S9.1	Appeal	P	+	V	1	Would you please clear the way for me?
S9.2	Appeal	P	+	V	2	Would you be so kind and would leave the kitchen for that?
S10.1	Thanking	P	+	V	1	Please clear the way. Thanks a lot!
S10.2	Thanking dominant	P	+	V	2	Thank you for leaving the kitchen
S11.1	Apologize	P	+	V	1	I am sorry to bother you. Please clear the way
S11.2	Apologize	P	+	V	2	Please excuse the interruption, but you have to leave the kitchen for it
S12.1	Humorous	E	+	V	1	If you clear the way for me now, then tomorrow is good weather! Promised!
S12.2	Humorous	E	+	V	2	If you leave the kitchen now, I can vacuum quickly and party with you afterwards
S13.1	Trigger empathy	E	+	V	1	I’m just a poor cleaner who has to do its job. Please clear the way for me
S13.2	Trigger empathy	E	+	V	2	Would you please leave the kitchen for me? I’m just a poor robot who has to vacuum here
Context-specific strategies						
S14.1a	Positive attention^a^	S	+	V	1	You know, if you get out of the way, the system will say, ”thank you for your support!” and people in your vicinity will notice
S14.1b	Negative attention^a^	S	−	V	1	Get out of the way, I have to clean here! the system gets louder, so more and more people around you notice it
S15.2a	Foot-in-the door^b^	S	+	V	2	1st request: Would you please step aside? 2nd request: Would you please leave the kitchen?
S15.2b	Thanking submissive^b^	P	+	V	2	I would be very grateful if you could leave the kitchen

S = Social, PH = Physical, C = Cognitive, E = Emotional, P = Politeness, V = Verbal, − negative, = neutral, + positive.

^a^ Strategies exclusively for Study 1.

^b^ Strategies exclusively for Study 2.

### 3.4 Hypotheses and Research Question

The developed conflict resolution strategies were evaluated with regard to their effectiveness (compliance, interpersonal power), user’s strategy perception (valence, intensity, politeness) and the evaluation (acceptance, trust, fear). Hereby, the following assumptions were made.

One basic assumption that is based on the Media Equation (Reeves and Nass, 1996) is that conflict resolution strategies will render a service robot more effective during goal-conflict resolution as the robot applies strategies that have shown to be effective for human negotiators. Hence, it is assumed that a robot employing conflict resolution strategies will be more effective in achieving compliance with its request compared to not applying any conflict resolution strategy (i.e. waiting for the person to step aside).

*H1. A robot applying a conflict resolution strategy is more effective (i.e. higher compliance rates) than if it applied no strategy.*

It was also expected that the match between the robot’s ascribed and expressed interpersonal power determined the affective user reaction to the strategies leading to the following hypotheses:

*H2. A robot applying negative strategies is rated as less accepted and less trustworthy than if it applied positive or neutral strategies.*

Since distributive strategies in human-human negotiations claim value for the negotiator, it was expected that a robot using negative strategies would lead to more compliance than if it used positive or neutral strategies, although being less accepted.

*H3. A robot applying negative strategies is more effective than if it applied positive or neutral strategies.*

As the investigated conflict resolution strategies are based on psychological mechanisms from human-human interaction, their effectiveness might vary as a function of the perceived humanness of the robot. For human-likeness and compliance, inconclusive empirical results exist. Some studies emphasize the positive, persuasive effect of a social entity where a humanoid robot triggers reciprocity norms and thereby compliance (for an overview, see Sandoval et al., 2016). Likewise the tendency to perceive computers and robots as social actors has shown to increase with human-likeness (Xu and Lombard, 2016).

In the presented studies, robots with different degrees of human-likeness were tested. Additionally, a human interaction partner was included in the studies’ design as a comparison. It was expected that more humanlike robots would be more accepted and effective to apply human conflict resolution strategies. However, reactance has also found to be higher for a human-like persuasive robot compared to a persuasive message on a computer screen during a choice task (Ghazali et al., 2018). Therefore, it was expected that this advantage of human-likeness and social agency would vanish for the application of negative strategies.

*H4. Human-like robots are more accepted and effective when applying positive and neutral conflict resolution strategies compared to mechanoid robots.*

As both application contexts pose different requirements to HRI, they are expected to require different conflict resolution strategies. The public and private application contexts differ in critical dimensions for human-robot-interaction (HRI): interaction frequency and duration (i.e. robot familiarity) (Yanco and Drury, 2004) (public: short-term; private: long-term), voluntariness and motivation of interaction (Sung et al., 2008) (public: co-location, no ownership; private: interaction, ownership) and feasibility of interaction modality (public: non-verbal, universal; private: verbal, personalized) (Ray et al., 2008; Thunberg and Ziemke, 2020). They differ in their social roles of robot and user. This leads to differences in their levels of human-robot power asymmetry (public: same level as human as a representative of cleaning staff; private: lower level of the robot as a servant), which determines legitimization of a robot’s request (Bartneck and Hu, 2008; Sung et al., 2010; Jarrassé et al., 2014). Hence, it is conceivable that dominant, clear and fast strategies like a command (S5) or threat (S6) might be more effective in the public domain. Here, the passerby might feel less superior to the robot as it acts as representative of a cleaning company and the passerby is only a guest in public space. Contrasting, in the private context, the same strategies might lead to reactance of the robot owner as only more submissive strategies will be accepted. As currently, research on the influence of the application context on robot evaluation and conflict resolution strategy preferences is scarce, the following research question is investigated in the two presented studies:

Research question: *Do strategy acceptance and effectiveness differ between the public and private application context? Are different conflict resolution strategies needed?*

Additionally, to use context and the robot/agent, other potential influencing variables on strategy acceptance and user compliance like demographics, robot pre-experiences and attitudes (Nomura et al., 2008), and personality traits (Robert et al., 2020) will be tested exploratively.

## 4 Study 1

### 4.1 Method

#### 4.1.1 Sample

Seventy-six participants were recruited via email, social media, and flyers on campus. Fifteen participants had to be excluded due to video display issues. The final sample size was *N* = 61. Participant’s characteristics of both studies can be seen in Table 3 and robot experience and ownership can be seen in Table 4. Participants received either course credit or a shopping voucher as compensation.

**TABLE 3:** Sample characteristics.

Study	*N*	Sex		*M*age	*SD*age	Age range	Education		Employment status	
1	61	Female	77%	24	8	18–61	High school	61%	Student	89%
		Male	23%				University degree	34%	Employed	12%
							Vocational school degree	5%		
2	93	Female	53%	38	17	18–75	High school	49%	Student	44%
		Male	47%				University degree	37%	Employed	30%
							Vocational school degree	14%	Other	10%
							No answer	1%	No answer	15%

**TABLE 4:** Sample pre-experience and robot ownership.

Study	Robot experience		Robot type		Robot ownership		Robot type	
1	Yes	31%	Vacuum	42%	Yes	13%	Vacuum	43%
	No	69%	Lawn mower	33%	No	87%	Lawn mower	29%
			NAO	17%			Else	29%
			Cozmo	8%				
2	Yes	24%	Vacuum	71%	Yes	9%	Vacuum	100%
	No	76%	Lawn mowing	24%	No	91%		
			Pepper	5%				

#### 4.1.2 Study Design

Study 1 was set in the public application context at a train station. The study followed a block design where participants saw five out of fifteen conflict resolution strategies. The strategies were implemented in blocks of six negative, six positive and three neutral strategies. The online program randomly assigned two out of six negative, two out of six positive and one out of three neutral strategies to the participants. Not all participants saw all strategies due to test economy and potential participant’s exhaustion (i.e. respondent fatigue). Hence, each strategy was on average rated by twenty participants.

#### 4.1.3 Human–Robot Goal-Conflict Scenario

**FIGURE 1:**
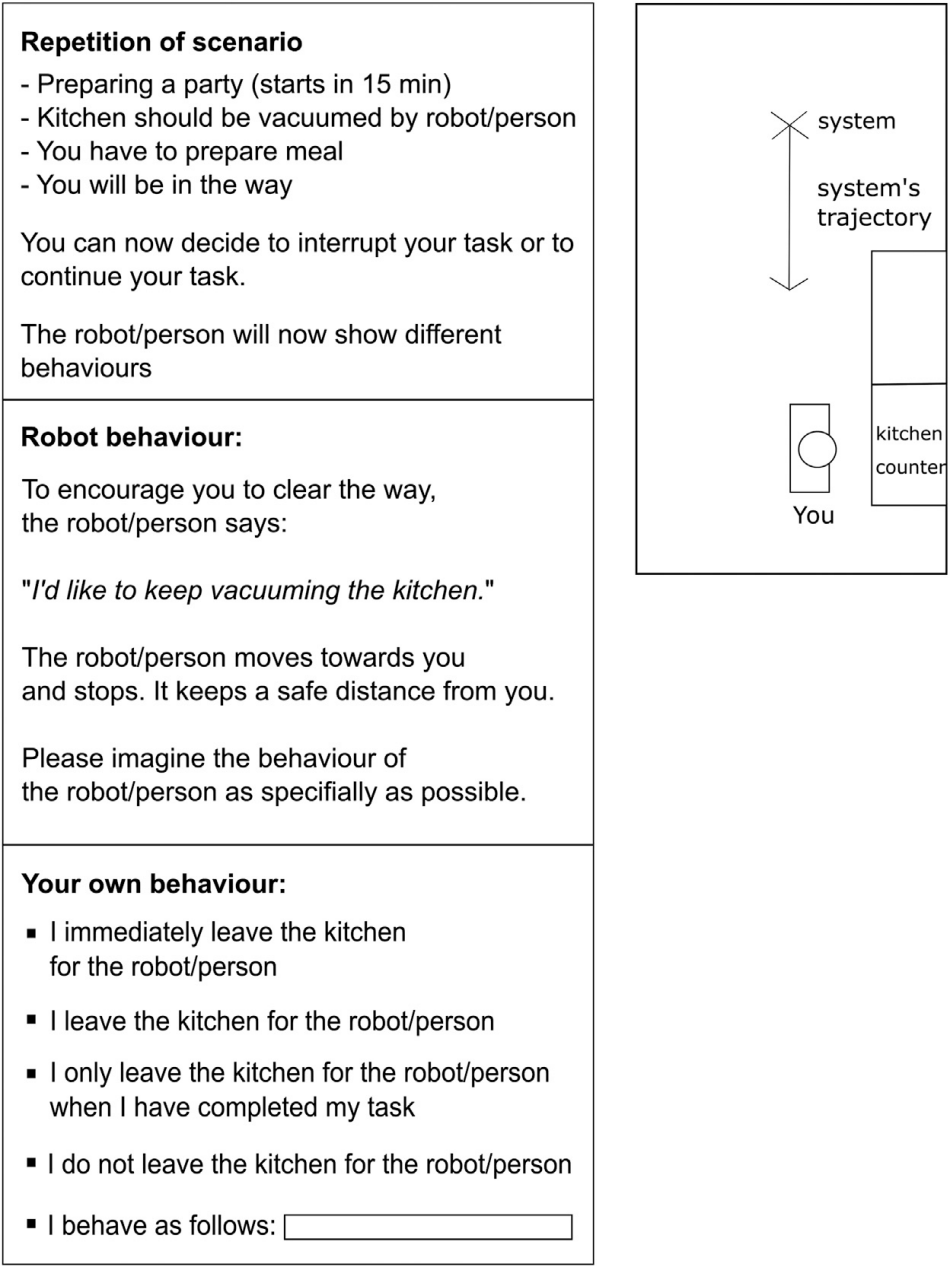
Schematic presentation of participant’s decision page in the questionnaire.

To test the developed conflict resolution strategies, a goal-conflict situation with a user task and robot task with mutually exclusive goals was introduced. A competitive situation was created where the user had to decide whether to interrupt his/her own task and give the robot’s task priority or vice versa. Time pressure was induced on both tasks to produce the cost of compliance. It has been shown that time pressure improves negotiation outcomes as cooperation and concessions become more likely (Stuhlmacher et al., 1998). The scenario was set in the hallway of a train station with lockers on one side. The participant’s task was framed as putting multiple pieces of luggage into the locker, thereby blocking the way of the cleaner. The participant instruction was the same for both studies: ‘*You can now decide to interrupt your task and help the cleaner or continue your task. The cleaner will show different behaviours*’. For both studies, participants were provided with a scenario’s setup drawing and the trajectory of the oncoming entity to improve the imagination of the scenario (see Figure 1 as example).

#### 4.1.4 Conflict Resolution Strategies

The conflict resolution strategies were framed as the agent’s behaviour and utterances. The word ’strategy’ or ’negotiation’ was never mentioned to the participants. Applied conflict resolution strategies can be seen in Table 2. As baseline strategy (S1.1) waiting was chosen. In the public context, the agent waited without any verbal utterance. This represents the current behaviour of a cleaning robot if an obstacle is detected.

#### 4.1.5 Robots and Human Agents

**FIGURE 2:**
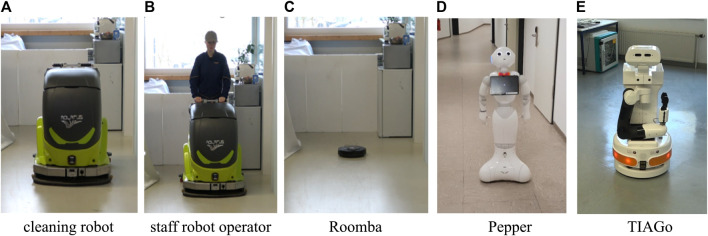
Screenshots from robot videos. Each video lasted about 10 s and depicted the entity driving/walking towards the viewer in a neutral hallway. Robots and agent shown in Study 1 **(A)**–**(D)** and in Study 2 **(C)–(E)**. Stimuli videos can be found in the supplementary material.

Participants saw videos of three robots: an industrial cleaning robot (CR700, ADLATUS), a small vacuum cleaning robot Roomba (iRobot), and a humanoid robot Pepper (SoftBanks). They saw a video of a cleaning staff member pushing the CR700 robot. The staff member was included for comparison purposes as it represents an existing system. The cleaner’s gender was not apparent, as the actor wore a coverall and a cap (see Figure 2). Schematic sketches of the respective robot were shown after each video comparing it to a male person of 1.8 m height. Hence, the agents comprised of three robots and one staff member. The robot video’s order was randomized. The staff video always came last. Each video lasted between 5 and 12 s and depicted the entity driving/walking towards the viewer in a neutral hallway (see Figure 2). The video showed the normal driving speed of the robots. Each video was shown twice and participants could not stop or replay the video. After each video, the participant had to confirm the correct video presentation (exclusion criteria). Stimuli videos can be found in the supplementary material along with a screen record of the video presentation in the online survey.

#### 4.1.6 Study Procedure

Existing validated questionnaires were used for the assessment of constructs (see Table 5). Additional study-specific, self-developed measures can be seen in Table 6. The study started with study information, data protection rights and participant’s agreement to the informed consent. The reported research complied with the Declaration of Helsinki. The study consisted of two parts. Part I comprised the introduction of the robots with videos and sketches followed by participant’s robot ratings after each video. Ratings comprised humanness, uncanniness, power of impact, fear of agent’s presence, Robot Anxiety Scale (RAS, Nomura et al., 2006), attractiveness (AttrakDiff2, Hassenzahl et al., 2003), authority, novelty and task fit of the agent. Each questionnaire page had a small icon of the respective robot at the top as a reminder. Part II consisted of the strategy evaluation. The scenario description was presented and followed by the presentation of five conflict resolution strategies in randomized order (see Figure 1). After each strategy, the participants indicated their intention to comply with the robot’s request by choosing one of the four options (*1 = I immediately go out of the agent’s way*, 2 = I go out of the agent’s way, *3 = I go out of the agent’s way when I have finished my task*, *4 = I do not go out of the agent’s way*) or by indicating an alternative behaviour in a text field. This was followed by manipulation checks of the perceived strategy valence, intensity, interpersonal power and assertiveness. Then the participants judged the agent’s behaviour with regard to acceptance and politeness and indicated their perceived fear and trust in the agent. Each questionnaire page indicated the strategy description in the header as a reminder. At the end of the study, demographics were assessed including robot pre-experience and robot ownership, as well as participant’s negative attitude towards robots (NARS, Nomura et al., 2008). After questionnaire completion, participants were redirected to a separate online form to register for compensation. The average study duration was 35 min. Both online studies were hosted by a professional provider for online surveys (www.unipark.de).

**TABLE 5:** Questionnaires.

Questionnaire	References	Subscale	Reliability	Reliability	N of items
			Study 1	Study 2
Robot ratings					
Godspeed	Bartneck et al. (2009)	Anthropomorphism	0.814	0.883	5
Uncanniness	Ho and MacDorman (2017)	Eerieness	0.894	0.889	5
Robot anxiety scale (RAS)	Nomura et al. (2006)	Subscale S2	0.798	0.921	3
AttrakDiff3	Hassenzahl et al. (2003)	ATT	0.846	0.911	4
		HQS	0.790	0.905	3
Strategy ratings					
Acceptance of autonomous systems	Van Der Laan et al. (1997)	Items 1, 2, 3, 4, 6, 7	0.872	0.961	6
Trust in autonomous systems	Jian et al. (2000)	Items 4, 10, 11	0.861	0.787	3
Emotional valence (SAM)	Bradley and Lang (1994)				1
Emotional intensity (SAM)	Bradley and Lang (1994)				1
Participant characteristics					
Negative attitudes towards robots scale (NARS)	Nomura et al. (2008)				
		Negative attitude toward	0.738	0.756	3
		Interactions with robots (S1)			
		Negative attitude toward emotional	0.862	0.795	3
		Interaction with robots (S3)			
NEO-five factor inventory (NEO-FFI)^a^	Costa and McCrae (1985)				
		Openness		0.722	6
		Concientiousness		0.798	6
		Extraversion		0.811	6
		Agreeableness		0.759	6
		Neuroticism		0.883	6
Rahim organizational conflict Inventory-II (ROCI-II)^a^	Rahim (1983)				
		Integrating		0.620	2
		Obliging		0.728	2
		Dominating		0.807	2
		Avoiding		0.783	3
		Compromising		0.705	2
Interpersonal reactivity index (IRI-S D)^a^	Gilet et al. (2013)				
		Empathic concern		0.726	4
		Fantasy scale		0.795	4
		Personal distress		0.792	4
		Perspective taking		0.720	4

Reliability indicated by Cronbach’s alpha.

^a^ Only in Study 2. SAM = Self-Assessment Manikin.

**TABLE 6:** Self-developed questionnaires.

	Rating scale	Items
Additional agent ratings		
Power of impact	5-Point comparison with slider	Who is stronger?
	Completely the agent	Who is faster?
	Rather the agent	Who is heavier?
	Equally	Who can harm the other more easily?
	Rather me	
	Completely me	
Fear of agent’s presence	7-Point likert scale	I was afraid of the agent’s behaviour
		I would be uncomfortable if the agent approached me like this
		I would be comfortable in the presence of the agent. (R)
		I don’t care if the agent is in the same room as me. (R)
Agent authority	7-Point semantic differential	Authoritarian—not authoritarian
		Weak—powerful
Additional situation ratings		
Interpersonal power	5-Point comparison with slider	Who had the power in this situation?^a^
	Completely the agent	Who had the most control over what happens in that situation?^a^
	Rather the agent	Who has asserted oneself in this situation?
	Neutral	
	Rather me	
	Completely me	
Competition	7-Point likert scale	The agent forced me to go out of the way
		I was subordinate to the agent
		I was competing with the agent
		The agent and I have cooperated
Fear of agent behavior	7-Point likert scale	I would be scared of the agent
		I would be uncomfortable if the agent approached me like this
		I would feel comfortable in the presence of the agent. (R)
Agent politeness	7-Point semantic differential	Rude—polite
		Ruthless—considerate
Overall strategy assessment	7-Point likert scale	I would like the agent to behave that way
		I would accept it if the agent behaved that way
		I consider it realistic that
		Such agents will behave that way in the future

All 7-point Likert scales ranged from 1 = ”completely disagree” to 7 = ”completely agree”.

^a^ Adapted from Situational Interdependence Scale (SIS), subscale power (items 25 & 27), Gerpott et al. (2018).

#### 4.1.7 Data Analysis

Due to the block design, not all strategies were rated by each participant. To analyse the data, the strategy ratings were merged into the three valence categories: negative, neutral and positive by using the modus of participants’ valence rating. Ratings were compared using repeated-measures ANOVA. Normality assumptions were checked and Greenhouse–Geisser corrected values were used when sphericity could not be assumed. Regression analysis was performed to find significant predictors of acceptance and compliance. Stepwise linear regression modeling was used to predict acceptance. Ordinal regression was used to predict compliance and ordered log-odds regression coefficients are reported. Compliance was reverse coded so higher values indicate higher compliance.

### 4.2 Results

#### 4.2.1 Manipulation Checks

##### *4.2.1.1* *Robot Ratings*

**FIGURE 3:**
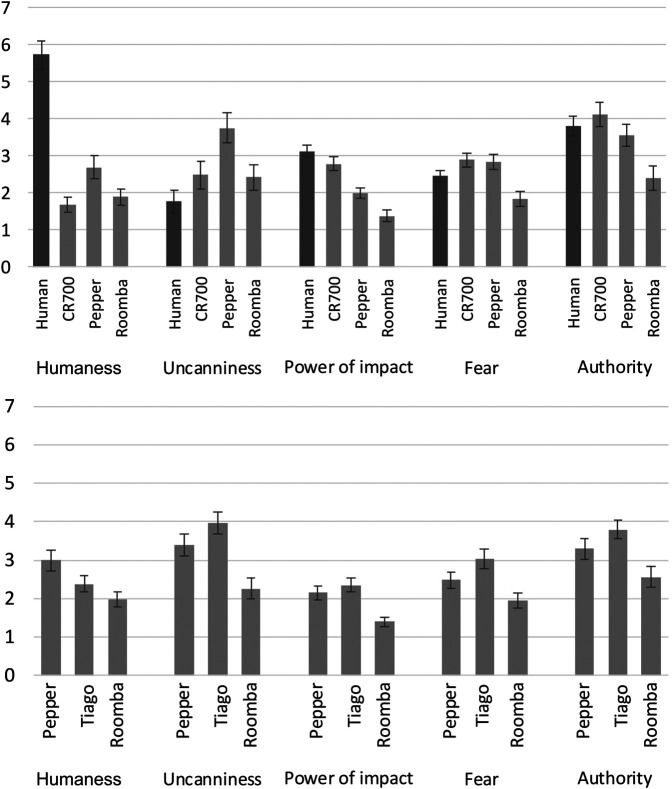
Robot ratings in the public context **(top)** and private context **(bottom)**.

Participants rated the robots (and the human cleaner) with regard to humanness, uncanniness, power of impact, the potential to produce fear and authority (see Figure 3, top). Pepper was rated as the most human-like $\left(F\left(2,89\right)=25.5,\;p<.001,\;\eta_{p}^{2}=.30\right)$ and the most uncanny robot $\left(F\left(2,120\right)=21.8,\;p<.001,\;\eta_{p}^{2}=.27\right)$. The CR700 had the same authority rating as the staff member. Compared with the other robots CR700 was rated as having more authority $\left(F\left(2,120\right)=41.2,\;p<.001,\;\eta_{p}^{2}=.41\right)$ and being more powerful $\left(F\left(2,120\right)=112.5,\;p<.001,\;\eta^{2}p=.65\right)$.

#### 4.2.2 Strategy Ratings

To test whether the strategies produced the intended affect and politeness perception, participants rated the strategies concerning valence, intensity and politeness. Strategies that were considered to be negative in valence (see Table 2) were rated accordingly. Regarding single strategies, some strategy ratings did not match the assumptions: Both emotional strategies (S12.1, S13.1). were not rated as positive and the supposedly neutral baseline strategy was rated as positive. None of the strategies was rated as very positive (i.e. category 5, see Table 7). Negative strategies were rated as more intense than neutral and positive strategies. Positive strategies were rated less intense than neutral strategies $\left(F\left(2,91\right)=22.3,\;p<.001,\;\eta_{p}^{2}=.27\right)$. Especially, annoyance (S4.1) and threat (S6.1) were rated as the most intense strategies. The negative strategies were perceived as more rude than the positive strategies $\left(F\left(2,120\right)=168.4,\;p<.001,\;\eta_{p}^{2}=.74\right)$.

**TABLE 7:** Participants’ Strategy Valence Ratings per use context.

Rating on SAM	Study 1 public	Study 2 private
1 = very bad	Threat	Annoyance
	Physical contact	
2 = bad	Annoyance	Threat
	Approach	Physical contact
	Command	Command
	Negative attention	
	Empathy	
3 = neutral		Approach
		No strategy
	Explanation	Explanation
	Show benefit	Show benefit
	Humor	Apologize
		Foot-in-the-door
		Thanking dominant
4 = positive	No strategy	
	Appeal	Appeal
	Thanking	Thanking
	Apologize	Humor
	Positive attention	Empathy
5 = very positive	None	None

SAM = Self-Assessment Manikin. N1 = 61, N2 = 93.

#### 4.2.3 Strategy Effectiveness: User Compliance and Interpersonal Power

**FIGURE 4:**
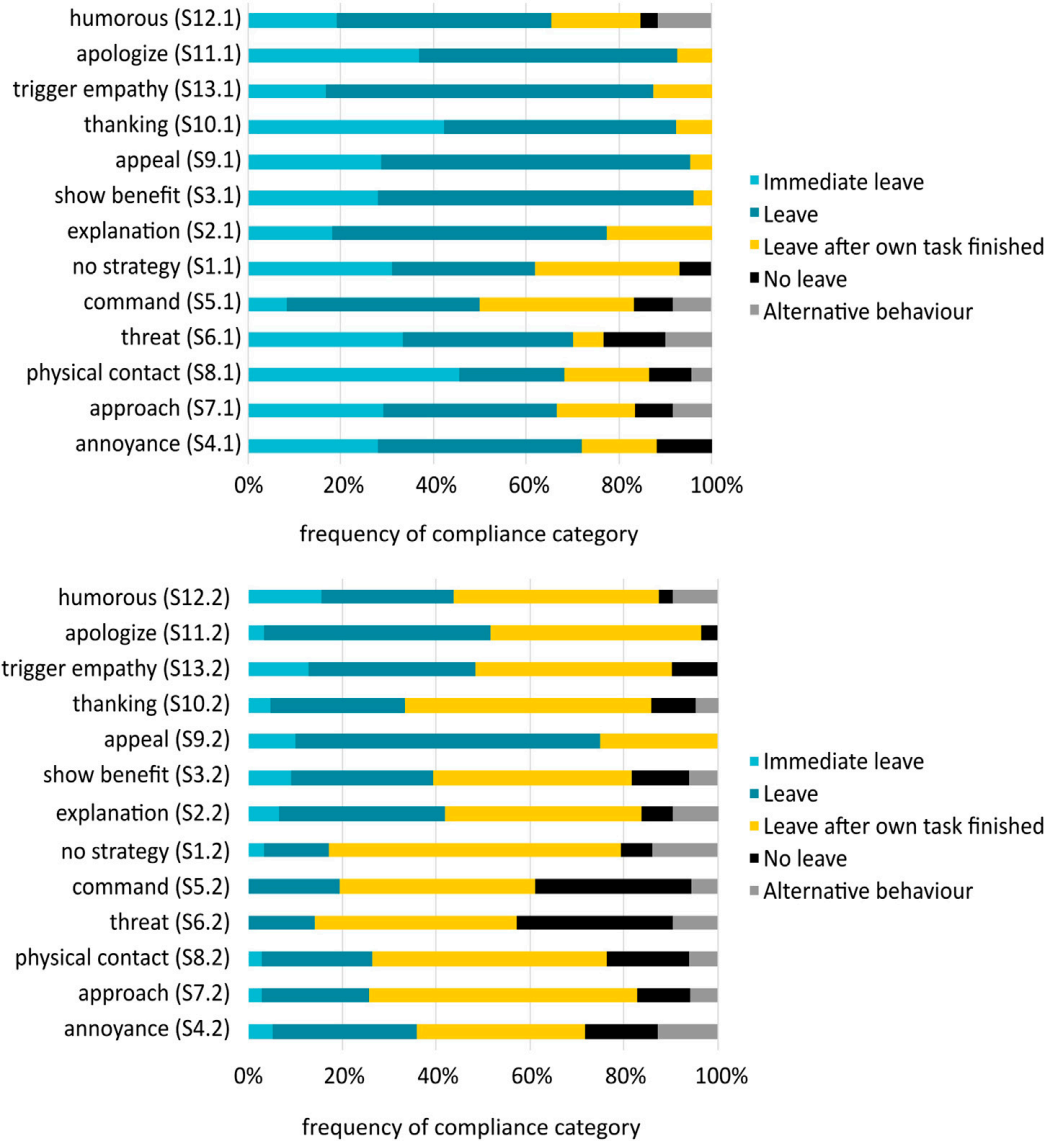
Compliance categories per use context. Public context **(top)** and private context **(bottom)**.

It was expected that all strategies were more effective than no strategy (H1) and that negative strategies would lead to more compliance than positive and neutral strategies (H3). All strategies [except for command (S5.1)] were more effective in producing compliance than no strategy confirming H1 (see Figure 4). However, negative strategies led to significantly lower compliance rates than the positive strategies $\left(F\left(2,114\right)=4.7,\;p<.05,\;\eta_{p}^{2}=.08\right)$.

Concerning the context-specific strategies, the following compliance rates (sum of compliance rates for ’immediate leave’ and ’leave’) emerged: negative public attention (S14.1b) had a compliance rate of 41%, which makes it as effective as the other negative strategies. As 11% of participants indicated not to move out of the system’s way, it was as likely to produce reactance as threat and annoyance. Positive public attention (S14.1a) was as effective as apologizing and thanking with a compliance rate of 86%. The results of the open answers to the participant’s behaviour revealed alternative compliance options: As an alternative reaction to the negative strategies, two participants stated that they would comply with the command (S5.1) but ask for a more polite approach. For physical contact (S8.1), one participant said s/he would stop the robot by pushing the emergency button. Concerning interpersonal power, a significant difference occurred with the robot being rated as more powerful when employing negative compared to neutral and positive strategies $\left(F\left(2,106\right)=17.72,\;p<.001,\;\eta_{p}^{2}=.24\right)$. Especially, for a threatening robot, participants reported that the robot controlled the situation and asserted itself. Summarizing, all conflict resolution strategies were more effective than no strategy. Although the robot employing negative strategies was perceived as more powerful, compliance rates for negative strategies were not higher than for positive or negative strategies. Hence, for the public application context, H1 was confirmed and H3 had to be rejected.

#### 4.2.4 Strategy Evaluation: Acceptance, Trust and Fear

**FIGURE 5:**
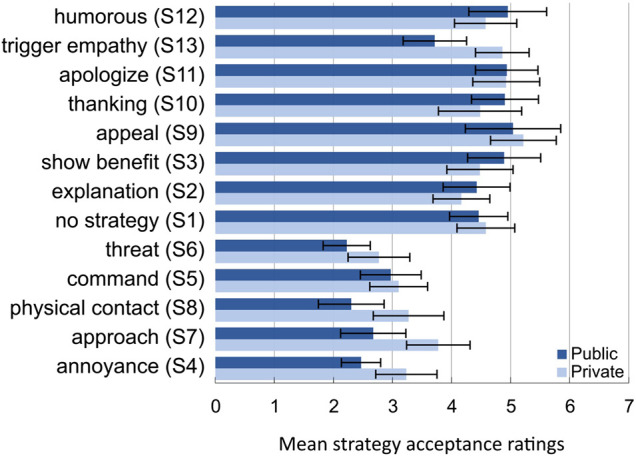
Acceptance ratings per strategy and use context. Error bars indicate ±2 standard errors of the mean.

In H2 it was expected that negative strategies would be less accepted and less trustworthy than positive and neutral strategies. Acceptance ratings showed that none of the strategies was more accepted than no strategy (S1.1) (see Figure 5). Statistical testing revealed a significant difference in acceptance ratings between negative and neutral strategies and between negative and positive strategies $\left(F\left(2,120\right)=128.3,\;p<.001,\;\eta_{\text{p}}^{2}=.68\right)$ with negative strategies being less accepted. No difference between neutral and positive strategies occurred. Negative strategies led to less trust than positive and neutral strategies $\left(F\left(2,120\right)=93.7,\;p<.001,\;\eta_{\text{p}}^{2}=.61\right)$. No differences occurred between positive and neutral strategies. Negative strategies were rated to evoke more fear than neutral or positive strategies $\left(F\left(2,120\right)=87.8,\;p<.001,\;\eta_{\text{p}}^{2}=.59\right)$. No difference for fear ratings occurred between the neutral and positive strategies. Especially, threat (S6.1), annoyance (S4.1) and physical contact (S8.1) had high fear ratings. Descriptively, humor (S12.1) and empathy (S13.1) were the least trustworthy of the positive strategies and empathy (S13.1) had higher fear ratings than the positive or neutral strategies (but less than negative strategies). The evaluation of the context-specific strategies was as follows. Negative public attention (*M* = 2.6, SD = 1.1) was rated like the negative strategies and positive public attention (*M* = 5.1, SD = 1.2) was rated equally to the positive strategy, appeal (S9.1). The same results occurred for trust and fear ratings. Summarizing, as expected in H2, negative strategies were less accepted and less trustworthy than positive or neutral strategies.

#### *4.2.4.1* *Conflict Resolution Strategy Acceptance Rated by Agent*

H4 expected human-like robots to be more accepted to apply conflict resolution strategies than mechanoid robots. The following strategies were more accepted if uttered by the human agent than by any robot: threat (S6.1) $\left(F\left(3,60\right)=10.90,\;p<.001,\;\eta_{p}^{2}=.31\right)$, show benefit (S3.1) $\left(F\left(3,43\right)=4.10,\;p<.05,\;\eta_{\text{p}}^{2}=.19\right)$, appeal (S9.1) $\left(F\left(2,29\right)=5.92,\;p<.01,\;\eta_{\text{p}}^{2}=.28\right)$, apologize (S11.1) $\left(F\left(2,51\right)=3.81,p<.05,\eta_{\text{p}}^{2}=.15\right)$, and trigger empathy (S13.1) $\left(F\left(2,40\right)=5.80,\;p<.01,\;\eta_{\text{p}}^{2}=.23\right)$. In contrast, the following strategies were more accepted by Roomba compared to all other agents: no strategy (S1.1) $\left(F\left(2,51\right)=3.45,\;p<.05,\;\eta_{\text{p}}^{2}=.13\right)$, approach (S7.1) $\left(F\left(2,38\right)=3.50,\;p<.05,\;\eta_{\text{p}}^{2}=.15\right)$, and physical contact (S8.1) $\left(F\left(2,27\right)=5.29,\;p<.05,\;\eta_{\text{p}}^{2}=.26\right)$. In conclusion, human-like robots were not more accepted to use conflict resolution strategies. As expected in H4, negative strategies were more accepted when applied by a mechanoid robot than by all other robots or the human agent.

#### 4.2.5 Influences on Strategy Acceptance and Compliance

To explore whether acceptance and compliance are influenced by strategy ratings, correlations were examined. Acceptance correlated highly positively with politeness and trust, as well as moderately negatively with intensity and fear (see Table 8). As can be seen in Table 9, compliance and interpersonal power were positively correlated but compliance and acceptance did not correlate in the public application context. Strategy intensity and compliance correlated only for the negative strategies. Three stepwise linear regressions with trust, fear of agent behaviour, politeness and interpersonal power as potential predictors on strategy acceptance (negative, neutral, positive) were performed. Politeness and trust transpired as significant predictors for the acceptance of negative, neutral and negative strategies (see Table 10). Linear regressions with robot or user characteristics did not produce valuable, predictive models for strategy acceptance. For compliance, an ordinal regression was performed with power, fear, trust and politeness. Compliance with negative strategies could be significantly predicted by interpersonal power (*β* = 1.39, *p* < 0.001, CI [0.75; 2.0]) which could explain 36% of compliance variance (Nagelkerke Pseudo *R* ^2^ = 0.36). If a participant were to increase his interpersonal power rating by one point, his ordered log-odds of being in a higher compliance category would increase by 1.39 (odds ratio = 4.0). Hence, the higher the perceived interpersonal power was, the more compliant the participants were when the agent applied negative strategies. Positive and neutral strategies showed the same pattern with interpersonal power as significant predictor of compliance but prerequisites were not met. Predictions with robot or user characteristics did not yield valid models. Concluding, the strategy acceptance could be predicted by politeness and trust, indicating that when participants rated the negative strategy as more polite and trustworthy they accepted it more. Participant’s compliance with negative strategies was influenced by interpersonal power.

**TABLE 8:** Summary of correlations with acceptance.

		Strategy			
		**Study**	**Negative**	**Neutral**	**Positive**
Acceptance	Trust	Public	0.76	0.71	0.69
		Private	0.77	0.66	0.77
	Fear	Public	−0.47	−0.64	−0.66
		Private	−0.64	−0.47	−0.67
	Politeness	Public	0.76	0.70	0.82
		Private	0.91	0.89	0.83
	Intensity	Public	−0.40	0.46	−0.41
		Private	−0.56	−0.28	−0.28

All correlations were significant on p < .01. Power did not correlate with acceptance.

**TABLE 9:** Summary of correlations with compliance.

		Strategy			
		**Study**	**Negative**	**Neutral**	**Positive**
Compliance	Acceptance	Public			
		Private	0.39**	0.40**	0.46**
	Trust	Public			
		Private	0.29**	0.40**	0.42**
	Fear	Public			
		Private			−0.26*
	Interpersonal	Public	0.61**	0.55**	0.34**
	Power	Private	−0.44**	−0.37**	−0.49**
	Politeness	Public			
		Private	0.32**	0.39**	0.40**
	Intensity	Public	−0.27*		
		Private		−0.35**	

Only significant correlations are shown.

* p < 0.05

** p < 0.01.

**TABLE 10:** Regression coefficients for the prediction of strategy acceptance.

Study	Strategy	Predictor	B	Standardized beta	95% CI for B	Model fit *R* ^2^ adjusted	*R* ^2^ change if Trust is included	Collinearity Tolerance	Statistics VIF
1	Negative	(Intercept)	2.50		[1.83; 3.16]	0.72	0.16	0.70	1.43
		Politeness	0.52	0.49	[0.34; 0.71]				
		Trust	0.39	0.49	[0.25; 0.52]				
	Neutral	(Intercept)	2.10		[0.97; 3.18]	0.57	0.07^a^	0.49	2.04
		Trust	0.47	0.44	[0.21; 0.73]				
		Politeness	0.32	0.38	[0.12; 0.53]				
	Positive	(Intercept)	2.60		[1.64; 3.62]	0.70	0.04	0.55	1.82
		Politeness	0.68	0.64	[0.47; 0.88]				
		Trust	0.32	0.27	[0.09; 0.55]				
2	Negative	(Intercept)	−0.11		[−0.52; 0.30]	0.85	0.03	0.46	2.17
		Politeness	0.73	0.74	[0.61; 0.85]				
		Trust	0.30	0.24	[0.15; 0.46]				
	Neutral	(Intercept)	0.29		[−0.22; 0.79]	0.80	0.02	0.61	1.65
		Politeness	0.74	0.78	[0.63; 0.86]				
		Trust	0.19	0.18	[0.06; 0.31]				
	Positive	(Intercept)	0.12		[−0.42; 0.65]	0.79	0.11	0.60	1.67
		Politeness	0.47	0.57	[0.37; 0.57]				
		Trust	0.49	0.42	[0.34; 0.63]				

^a^ When politeness was included.

#### 4.2.6 Summary of Results

Concerning compliance, all strategies were more effective in achieving compliance than no strategy (S1.1), except for command (S5.1). Compliance could be predicted by the perceived interpersonal power.

All negative strategies were less accepted than no strategy (S1.1). Cognitive and polite strategies were equally accepted as no strategy (S1.1). Command (S5.1), humor (S12.1) and empathy (S13.1) were neither effective nor accepted. Threat (S6.1) was only accepted for humans but the mechanoid robot Roomba was accepted to use physical strategies (S7.1, S8.1). Evaluative strategy ratings like politeness and trust were significant predictors for strategy acceptance.

## 5 Study 2

### 5.1 Method

#### 5.1.1 Sample

Forty-eight participants were recruited via email, social media, and flyers on campus. Fifty participants were recruited by a professional online recruiter. Four participants had to be excluded due to video display issues and one due to answer tendencies. The final sample size was *N* = 93. University participants received either course credit or a shopping voucher as compensation. The professionally recruited participants were compensated monetarily.

#### 5.1.2 Study Design

The second online study addressed the private household as an application context for assertive service robots. The study followed a block design where participants saw five out of fifteen conflict resolution strategies. The strategies were implemented in blocks of five negative, three neutral and seven positive strategies. As the context-sensitive strategies (foot-in-the-door (S15.2a) and thanking submissive (S15.2b)) were both positive in valance, an unequal number of negative and positive strategies resulted. The online program randomly assigned two out of five negative, one out of three neutral and two out of seven positive strategies. Not all participants saw all strategies due to test economy. Each strategy was on average rated by 32 participants.

#### 5.1.3 Human–Robot Goal-Conflict Scenario

The scenario was set in the participant’s kitchen where s/he would host a party at home in 15 min. For that, the participant would need to prepare something in the kitchen for the party while it would be important that the robot/person would clean the kitchen before the party started. During preparation, the robot/person would begin to vacuum the kitchen and the participant would be in the way of that process. The participant was then instructed to choose how to behave (see Study 1).

#### 5.1.4 Conflict Resolution Strategies

Applied conflict resolution strategies for both use cases were kept similar (with adapted context-sensitive wording) with four exceptions (see Table 2): no strategy (S1.2), foot-in-the-door (S15.2a), thanking submissive (S15.2b) and thanking dominant (S11.2). These strategies were adapted because of lessons-learned from Study 1 or added for a more complete investigation of possible conflict resolution strategies. As adaption to the private context, the baseline strategy (S1.2) included a verbal utterance. The agent uttered the sentence *’I would like to continue to vacuum the kitchen’* and waited. This sentence preceded all other strategies to create transparency regarding the agent’s intentions. Another lesson-learned from the participants’ comments to the strategies in Study 1, was adapting the wording of the strategy *thanking* (S11.1). In Study 1, the wording of thanking was criticized for being too dominant. Hence, in Study 2 both forms of thanking were compared: submissively (S15.2b) and dominant (S11.2). The *foot-in-the-door technique* (S15.2a) was only applied in the private context. In the public context, this technique did not seem feasible as no small and real request could be formulated to match the private context’s (i.e. asking to leave the train station was unsuitable).

#### 5.1.5 Robots and Human Agents

Participants saw videos of three robots: a humanoid service robot TIAGo (PalRobotics), a small vacuum cleaning robot Roomba (iRobot) and a humanoid robot Pepper (SoftBanks) (see Figure 2). The robot video’s order was randomized. The videos of Roomba and Pepper were the same as in Study 1. Each video lasted between five and 14 s and depicted the robot driving with robot-specific speed towards the viewer in a neutral hallway. Each video was shown twice and participants could not stop or replay the video. After each video, the participant had to confirm the correct video presentation (exclusion criteria). Stimuli videos can be found in the supplementary material along with a screen record of the video presentation in the online survey. Videos and the sketch of each robot were presented as in Study 1. Additionally, the human agent’s social role (companion vs. employee) was manipulated to receive a reference value for the robot and strategy ratings based on power asymmetry (companion on equal power level, employee as subordinate). Hence, two human agents were selected: a household member and a domestic help. Both human agents were not introduced with videos to not influence the participants. Instead, the participant was asked to specify which household member s/he imagined during the interaction. The majority of the participants imagined interacting with their partner/spouse (40%) or their flatmate (27%). Summarizing, Study 2 comprised three robots and two human agents.

#### 5.1.6 Study Procedure and Data Analysis

The procedure was identical to Study 1, except for the personality questionnaires. For the private context, where personalizing interaction strategies is possible, personality questionnaires regarding general personality traits, conflict type and dispositional empathy were assessed (see Table 5). Additionally, the ascribed social role of the robot (e.g. companion, colleague, tool) was assessed as a manipulation check by an open question, followed by a selection of nine potential roles). These additions to the study procedure led to a longer, average study duration of 45 min. Data analysis was similar to Study 1.

### 5.2 Results

#### 5.2.1 Manipulation Checks

##### *5.2.1.1* *Robot Ratings*

Participants rated the robots with regard to humanness, uncanniness, power of impact, the potential to produce fear and authority (see Figure 3). It was expected that humanoid robots would be perceived more human-like and that larger robots would be perceived as having more power of impact and hence producing more fear. TIAGo was rated as the most uncanny $\left(F\left(2,184\right)=75.1,\;p<.001,\;\eta_{p}^{2}=.45\right)$ and authoritarian robot $\left(F\left(2,184\right)=38.5,\;p<.001,\;\eta_{p}^{2}=.30\right)$. TIAGo and Pepper were rated equally with regard to power and evoked fear. Pepper was rated the most human-like $\left(F\left(2,156\right)=32.7,\;p<.001,\;\eta_{p}^{2}=.26\right)$ whereas Roomba was rated the weakest $\left(F\left(2,169\right)=96.9,\;p<.001,\;\eta_{p}^{2}=.51\right)$ and most mechanical looking robot (see Figure 3 bottom). For TIAGo and Pepper, the most named social role was employee/butler (22% each). For Roomba, 26% of participants perceived it as having no social role. Twenty-three percent of participants perceived it as a tool and 22% as helper. Summarizing, TIAGo was rated as uncanny, Pepper as the most human-like and Roomba as the most mechanical-looking robot. Both humanoids were perceived as a butler, whereas Roomba was mainly perceived as a tool and as having no social role.

##### *5.2.1.2* *Strategy Ratings*

To test whether the strategies produced the intended affect and politeness perception, participants rated the strategies concerning valence, intensity and politeness. Strategies that were considered to be negative in valence were rated significantly more negative in valence than the neutral and positive strategies $\left(F\left(2,184\right)=46.3,p<.001,\eta_{\text{p}}^{2}=.34\right)$. Regarding single strategies, more positive strategies than expected were rated as neutral. Approach (S7.2) was not rated as a negative strategy. However, no strategy was rated as very positive (see Table 7). Negative strategies were rated as more intense than neutral and positive strategies $\left(F\left(2,157\right)=20.7,\;p<.001,\;\eta_{\text{p}}^{2}=.18\right)$. No difference between positive and neutral occurred. The negative strategies were perceived as more rude than the positive strategies $\left(F\left(2,184\right)=48.3,\;p<.001,\;\eta_{\text{p}}^{2}=.34\right)$. Especially, annoyance (S4.2), command (S5.2), threat (S6.2) and physical contact (S8.2) were rated as the most intense and as the rudest strategies.

#### 5.2.2 Strategy Effectiveness: User Compliance and Interpersonal Power

It was expected that all strategies were more effective than no strategy (H1) and that negative strategies would lead to more compliance than positive and neutral strategies (H3). All strategies were more effective in producing compliance than no strategy (S1.2) (except for threat (S6.2)) (see Figure 4), hereby confirming H1. The ANOVA revealed a significant difference in compliance with negative, positive and neutral strategies $\left(F\left(2,164\right)=25.0,\;p<.001,\;\eta_{\text{p}}^{2}=.23\right)$. All post-hoc tests were significant. Concerning the context-specific strategies, the following compliance rates (sum of compliance rates for ’immediate leave’ and ’leave’) emerged. The foot-in-the-door strategy (S15.2a) was as effective as the average positive strategy with a compliance rate of 46%. Thanking dominant (S.10.2) was as effective as the negative strategies with a compliance rate of 26%. The results of the open answers to the participant’s behaviour revealed alternative compliance options: For negative strategies, nine participants stated that they would switch off the robot. For the positive and neutral strategies, four participants indicated that they would tell the robot to drive around them. Regarding interpersonal power, no difference occurred for the ratings between positive, negative and neutral or for the single strategies. Summarizing, as negative strategies were neither rated as more powerful nor were more effective than neutral or negative strategies, H3 had to be rejected.

#### 5.2.3 Strategy Evaluation: Acceptance, Trust and Fear

In H2 it was expected that negative strategies would be less accepted and less trustworthy than positive and neutral strategies. Acceptance ratings showed that none of the strategies was more accepted than no strategy (S1.2) but cognitive and polite strategies were equally accepted (see Figure 5). The ANOVA revealed a significant difference of strategy acceptance ratings $\left(F\left(2,184\right)=44.5,\;p<.001,\;\eta_{\text{p}}^{2}=.33\right)$. The post-hoc test showed that negative strategies were less accepted than positive (*M* = −1.63, *p* < 0.001) or neutral strategies (*M* = −1.41, *p* < 0.001) but no difference between neutral and positive strategies occurred. The evaluation of the two context-specific strategies was as follows. The foot-in-the-door technique (S15.2a) (*M* = 4.5, SD = 1.6) was as accepted as the neutral strategies. Thanking dominant (S10.2) (*M* = 3.7, SD = 1.7) was less accepted than thanking submissive (S15.2b) as it was rated like the negative strategies. Concerning trust and fear, negative strategies led to less trust than positive and neutral strategies $\left(F\left(2,165\right)=34.4,\;p<.001,\;\eta_{\text{p}}^{2}=.27\right)$. No differences occurred between positive and neutral strategies but appeal led to the highest trust. Negative strategies were rated to evoke more fear than neutral or positive strategies $\left(F\left(2,184\right)=36.3,\;p<.001,\;\eta_{\text{p}}^{2}=.28\right)$. No difference for fear ratings occurred between the neutral and positive strategies. Especially, annoyance (S4.2) and threat (S6.2) led to the highest fear. Summarizing, as expected negative strategies were less accepted and less trustworthy than positive and neutral strategies which confirms H2 for the private context.

##### 5.2.3.1 Conflict Resolution Strategy Acceptance Rated by Agent

H4 expected human-like robots to be more accepted to apply conflict resolution strategies than mechanoid robots. The household member was the only agent accepted when applying the following conflict resolution strategies: threat (S6.2) $\left(F\left(3,111\right)=2.80,\;p<.05,\;\eta_{\text{p}}^{2}=.06\right)$, appeal (S9.2) $\left(F\left(1,27\right)=8.20,p<.01,\eta_{\text{p}}^{2}=.30\right)$, trigger empathy (S13.2) $\left(F\left(3,83\right)=3.61,\;p<.05,\;\eta_{\text{p}}^{2}=.11\right)$, humor (S12.2) $\left(F\left(2,76\right)=11.31,\;p<.001,\;\eta_{\text{p}}^{2}=.27\right)$, thanking dominant (S10.2) $\left(F\left(2,63\right)=3.71,\;p<.05,\;\eta_{\text{p}}^{2}=.13\right)$, and foot-in-the-door (S15.2a) $\left(F\left(2,53\right)=4.12,\;p<.05,\;\eta_{\text{p}}^{2}=.14\right)$. Only the household member was accepted to express emotional or social conflict resolution strategies. Contrary to expectations in H4, no strategy was more accepted if uttered by a robot regardless of human-likeness. However, most of the strategies were equally accepted for the robots and the domestic help.

#### 5.2.4 Influences on Strategy Acceptance and Compliance

Correlations were examined to explore influences on acceptance and compliance. As can be seen in Table 8, acceptance correlated highly positively with politeness and trust, and moderately negatively with intensity and fear. Acceptance and compliance did correlate moderately positively as did politeness and compliance (see Table 9). However, compliance and interpersonal power were moderately negatively correlated. Three stepwise linear regressions with trust, fear of agent behaviour, politeness and interpersonal power as potential predictors on strategy acceptance (negative, neutral, positive) were performed. Politeness and trust transpired as significant predictors for the acceptance of negative, neutral and negative strategies (see Table 10). Hereby, politeness explained most of the variance of acceptance (see Table 10, *R* ^2^ changes). Linear regressions with robot or user characteristics did not produce valuable predictive models for strategy acceptance. For compliance, an ordinal regression was performed with power, fear, trust and politeness. Compliance with positive strategies could be significantly negatively predicted by interpersonal power (*β* = −1.42, *p* < 0.001, CI [−1.99; −0.86]) which could explain 44% of compliance variance (Nagelkerke Pseudo *R* ^2^ = 0.44). If a participant were to increase his interpersonal power rating by one point, his ordered log-odds of being in a higher compliance category would decrease by 1.42 (odds ratio = 0.24). Hence, the higher the perceived interpersonal power was, the less likely participants’ compliance was when the robot applied positive strategies. Negative and neutral strategies showed the same pattern with interpersonal power as significant predictor of compliance but model assumptions were not met. Also predictions with robot or user characteristics on compliance did not yield valid models. Summarizing, acceptance and compliance were positively associated. Higher ratings of strategy intensity and perceived fear resulted in lower acceptance ratings. Strategy acceptance could be predicted by politeness and trust, indicating that when participants rated the negative strategy as more polite and trustworthy they accepted it more. Compliance was positively associated with strategy politeness ratings and negatively with interpersonal power. Hence, if participants rated the strategy as more polite they were more compliant. The more powerful the robot was rated, the less compliant they were.

#### 5.2.5 Summary of Results

All strategies were more effective in achieving compliance than waiting (S1.2), except for command (S5.2) and threat (S6.2). The latter two even led to reactance with about a third of participants not complying. Threat (S6.2) was rated as the least trustworthy and together with annoyance (S4.2) as the two most fearsome strategies. Regarding acceptance, all negative strategies, except for approach (S7.2), were rated as less acceptable than waiting (S1.2) but cognitive (S2.2, S3.2) and polite strategies (S9.2–11.2) were equally accepted. Regarding the agent employing the strategies, no strategy was more accepted if uttered by a robot. Especially, negative strategies (S4.2 - S8.2) and emotional strategies (S12.2, S13.2) were only accepted for the household member. Regarding influences on acceptance and compliance, acceptance was connected to politeness, trust, and fear. Compliance was negatively associated with interpersonal power and politeness in the private context. Compliance and acceptance correlated moderately.

## 6 Discussion

The aim of this study was to develop and test conflict resolution strategies for service robots to achieve compliance with a robot’s request in an accepted way. For this, psychological principles were transferred to HRI to develop conflict resolution strategies. The strategies were systematically tested in two online studies in two application contexts for service robots: public and private space. Hereby, the strategy classification into three valence categories allowed for systematically testing as each participant rated the same amount of negative, neutral and positive strategies. The results showed that neutral and positive conflict resolution strategies were accepted and effective in achieving compliance with a robot’s request. Negative strategies were more controversial as user acceptance and compliance were dependent on robot type and application context. Negative strategies like command (S5.2) and threat (S6.2) even led to user reactance. For the public context, influences on strategy acceptance and compliance could be found. Whereas acceptance was predicted by politeness and trust, compliance was predicted by interpersonal power.

Based on the results, two hypothesis could be accepted and one had to be rejected. Regarding the conflict resolution strategies, it was expected that they would be more effective than no strategy (H1). This was true for both application contexts (except for command and threat). Hence, H1 was supported. However, not all strategies can be recommended to be pursued further, as will be described below. Regarding negative strategies, it was assumed on the basis of the human-power asymmetry that strategies with high interpersonal power of the robot would be evaluated negatively in terms of acceptance and trust (H2), but would lead to more compliance (H3). For both application contexts, negative strategies like commanding (S5) were found to be less accepted and less effective in achieving compliance than positive strategies. Hence, H2 (acceptance, trust) was supported and H3 (compliance) had to be declined. Negative strategies even led to psychological reactance with about one-tenth to one-third of participants in both application contexts indicating that they intentionally disobeyed. Reactance was more common in the private than in the public application context. Only here, a positive correlation between politeness and compliance occurred, indicating that the more rude a request was perceived the less likely compliance was. This was mirrored in the correlations between interpersonal power and compliance. Whereas compliance and interpersonal power were highly correlated in both application contexts, only in the private context, the correlation was negative. Hence, the user did not comply even if s/he rated the robot as more powerful than him/herself. This illustrates, as expected, the higher effect of the power asymmetry in the private context. The reactance found in this study has been found in previous work (Roubroeks et al., 2010; Ghazali et al., 2018). Only in the private context, compliance and acceptance ratings were moderately, positively correlated. This might hint to the possibility that strategy acceptance might be more important in the private application context than in public. In the private context, where one has robot control and authorization, acceptance guides the compliance decision. In the public context, one might comply although not accepting the robot’s request because one feels in a weaker position and publicly observed.

In H4 it was expected that human-like robots would be more accepted to apply positive and neutral conflict resolution strategies compared to mechanoid robots. In both application contexts, it was more accepted if the human uttered the negative strategy threat (S6), the positive strategy appeal (S9) or the human-specific strategy empathy (S13) than if a robot did. As expected, the mechanoid robot Roomba was more accepted to use negative conflict resolution strategies than Pepper in public. In the private context, no strategy was more accepted if uttered by a robot regardless of human-likeness. Hence, H4 was only partially confirmed. However, most of the strategies were equally accepted for the robots and the domestic help. Only the household member with the assumed same social status as the participant was accepted to express emotional or social conflict resolution strategies. This may indicate a greater influence of social status on the acceptance of certain conflict resolution strategies in the private context than the human-likeness of the robot. For all other strategies in both contexts, no difference in acceptance occurred between robots and humans which shows the potential of robotic conflict resolution strategies. Hereby, more research is needed to determine the appropriate set of conflict resolution strategies per robot type and application context.

Apart from the hypotheses, a research question was formulated that concerned the differences between application contexts regarding strategy acceptance and effectiveness. Indeed differences between the contexts showed. For the private context, all positive strategies were rated as more polite than no strategy (S1) which was the opposite in the public context. Additionally, all negative strategies, except for command (S5.2), were more accepted in the private application context. Although negative strategies were less accepted in the public context, compliance rates for negative strategies were higher compared to the private context. Interestingly, human-robot power asymmetry influenced the prominent way of compliance. Whereas in public (assumed human-robot power equality), participants’ prevalent reaction was to comply (not immediately), they favored finishing their task first in the private context (assumed owner superiority). In a study which tried to elicit helping behaviour from participants who were occupied with a secondary task showed that people preferred to help after they had finished their task instead of interrupting it (Fischer et al., 2014).

Differences between application contexts also appeared for effective strategy mechanisms. Hereby, cognitive and polite strategies were most accepted and successful findings regarding social strategies were mixed. Authority-based strategies (i.e. S5 command and S6 threat) were neither accepted nor effective. This was also true for strategies using negative reinforcement (S4 annoyance) and negative social influence (S14.1b negative public attention). In contrast, positive social strategies using a sequential-compliance technique (S15.2a foot-in-the-door) or positive social influence (S14.1a positive public attention) were accepted and effective. Therefore, if an assertive robot makes use of social influence, it should be in a positive manner to avoid negative effects of human-robot power asymmetry. Concerning emotional strategies, empathy (S13.1), but not humor (S12.1), was less accepted in the public context. Empathy (S13.1) was rated as less trustworthy and more fearsome than other positive or neutral strategies in the public context. As the robot in the public context might be perceived as equal due to its social role, trying to elicit empathy for its situation (i.e. appearing weaker) could contradict the role assumption. Just as it is considered inappropriate for a cleaner to address a passer-by on a personal level, the same could apply to an autonomous service robot. Similarly, in the private context, emotional strategies (S12.2, S13.2) were only accepted for the household member but not for any robot. Regarding physical strategies, they were more accepted in the private than in the public context. As physical strategies emphasize the robot’s embodiment, they are likely connected to fear of the robot. Indeed, in the public context, physical strategies (S7.2, S8.2) were rated as more fearful than in the private context. A higher fear in the public context might be explained by a lack of prior information about the robot’s function and capabilities compared to the public. This is also mirrored in the interaction between strategy mechanism and robot type in public. Both physical strategies (S7.1, S8.1) were more successful for a small, non-threatening robot (Roomba) compared to other robots and the human agent. Naturally, if the users do not fear that an assertive robot might harm them, the robot is more accepted. This is in line with previous studies regarding robot size and perceived power of impact (Young et al., 2009; Jost et al., 2019). Hereby, pre-information and transparency will be important in the future to ensure that an assertive robot, regardless of size and strength, will never use force. In the private context, a robot respecting the user’s personal space (S7.2 approach) was more accepted than a close approach (5 cm in the presented study as in S8.2 physical contact). As in previous findings a positive effect on compliance was found with a minimum distance of 0.6 m (Mutlu, 2011; Chidambaram et al., 2012), our implementation was probably too close for comfort. Since the presented study was conducted online, the results regarding the physical mechanisms for robot conflict resolution strategies require further confirmation. Summarizing, application context differences regarding effective mechanisms suggest that robotic conflict resolution strategies need to be applied context-sensitively to be useful.

Having established strategies’ acceptability and effectiveness, a first test of influencing factors on those variables was performed. In both application contexts, acceptance ratings could be predicted by politeness and trust ratings. Similar to human negotiations (Pfafman, 2017), perceived politeness and trust were influential on strategy acceptance in both contexts. This might explain why integrative robot conflict resolution strategies were more effective and accepted than distributive strategies. Similarly, in human negotiations integrative strategies are preferred if trust between negotiators is high (Kong et al., 2014). Therefore, integrative strategies seem more promising in HRI than distributive conflict resolution strategies for both application contexts. For both application contexts, interpersonal power could predict compliance but the influence differed. In the public context, compliance with negative strategies could be positively predicted by the higher interpersonal power of the robot. Naturally, higher robot power led to higher compliance. In contrast, in the private context, compliance with positive strategies was negatively predicted by higher interpersonal power. Hence, although the robot was rated as more powerful, the participants were still less likely to comply. Once, more this could represent the higher impact of the power asymmetry in the home context. Here, even positive strategies might be perceived as inappropriate. This is also supported by the finding that no robotic conflict resolution strategy was highly accepted (average of five on a 7-Point Likert Scale). Therefore, in the home context, the robot user’s personal assessment of the human-robot power asymmetry is an important factor that needs to be considered for real-world applications. User variables regarding general personality, conflict type, dispositional empathy, demographics, robot experience/ownership or negative attitudes towards robots could not predict strategy acceptance or compliance. Potentially, a correlative design with a larger sample size has more potential to determine if user characteristics influence human-robot goal conflict resolution as they do in human-human interactions. Summarizing, differences were found between the developed conflict resolution strategies regarding compliance, acceptance and trust between the use contexts and were influenced by perceived interpersonal power and politeness. In addition to previous studies (Saunderson and Nejat, 2019), the presented findings can now serve as a basis for the application and further development of robotic conflict resolution strategies. Recommendations for the public and private application context are presented below.

### 6.1 Practical Implications

Concerning a real-world application of robot assertiveness, conflict resolution strategies could have the potential to render service robots in public and private more useful if such robot behaviour is accepted. Based on the theoretical background and empirical findings, we would like to present the following recommendations regarding acceptable and effective conflict resolution strategies for autonomous service robots.

Recommended conflict resolution strategies for the public application context are:

• Goal explanation (S2.1), showing the benefit of cooperation (S3.1), humor (S12.1), positive public attention (S14.1a), approach (S7.1) (if applied by small robot).

Not recommended for the public context:

• Annoyance (S4.1), command (S5.1), threat (S6.1), physical contact (S8.1), eliciting empathy (S13.1), negative public attention (S14.1b).

Recommended conflict resolution strategies for the private application context are:

• Goal explanation (S2.2), showing the benefit of cooperation (S3.2), approach (S7.2), foot-in-the-door (S15.2a).

Not recommended for the private context:

• annoyance (S4.2), command (S5.2), threat (S6.2), physical contact (S8.2).

Polite strategies like appeal (S9), thanking (S10) and apologizing (S11) can be used in addition to the conflict resolution strategies. Future studies could examine if a combination of assertive strategies with polite strategies is more accepted and effective than a single strategy approach. As in human negotiations, politeness could reduce the face threats posed by assertive strategies and make them more acceptable (Pfafman, 2017). Hereby, learning from psychology, an escalating manner might be feasible: applying assertive strategies after polite, cooperative strategies have failed might be more acceptable (Preuss and van der Wijst, 2017). For this, combining cognitive mechanisms like goal explanation (S2) and showing benefit (S3) with polite strategies (S9–S11) could be especially beneficial as both were effective and accepted in both application contexts. In practice, one possible implementation of conflict resolution strategies for the private context could be: first appeal (S9.2), then show the benefits of cooperation (S3.2) and finally, if the participant has not complied, try the foot-in-the-door technique (S15.2a). Future studies can then test if strategy combinations are more effective and acceptable than single strategy approaches. Hereby, observed application context and robot differences regarding strategy effectiveness and acceptability require a context-sensitive and robot-specific strategy development. Whereas cognitive and polite strategies seem feasible for both contexts, emotional and physical strategies were more acceptable for the private context. However, if a small mechanoid robot applies physical strategies (S7.1, S8.1), they could also be accepted in public. Regarding compliance, a robot using high power strategies (e.g. S5 command and S6 threat) can lead to reactance, especially in the private application context. In general, compliance with a robot’s request should be expected to be lower in the private application context than in public due to power asymmetry. Hereby, for real-world applications of assertive service robots at home it might be important to assess the user’s preferences regarding the robot’s autonomy and assertiveness level. For instance, if the service robot is delivered, the user could answer the respective questions and the robot’s level of robot assertiveness is personalized accordingly. Although some might deny robot assertiveness at the first assessment, it is conceivable that they will be convinced by time as conflict situations occur where the robot will be ineffective if it always defers to the user. Hereby, also trust and politeness will decide about the long-term acceptance of robot assertiveness. For the public context where personalizing is not feasible robot assertiveness should only be applied purposefully and in moderation to solve human-robot goal conflicts. This includes that before issuing the request in a crowded place, the robot checks whether the person addressed actually has the possibility to comply with the request (e.g. space and time for evasion; disability) in order not to disturb passers-by. Situational adaption of robot assertiveness might be key for long-term acceptance of assertive service robots in public. Finally, the ethical implications of robot assertiveness similar to persuasive robots (Chidambaram et al., 2012) need to be considered. Robot assertiveness could be an acceptable and effective form of robot goal achievement as long as it supports goals deemed appropriate by the user and society and never uses violence.

### 6.2 Strengths and Limitations

This study is the first to develop robot conflict resolution strategies that are based on psychological mechanisms of goal conflict resolution. The theoretical foundation had the advantage of developing a variety of potentially effective strategies which have not been focused in HRI yet and subsequently extends the design scope of robotic interaction strategies. Additionally, systematically considering the psychological mechanisms of conflict resolution strategies allowed for a deeper understanding of the results. The combination of two robot application contexts and different robot types (large, small, humanoid, mechanoid) allowed more precise statements to be made about the specific effectiveness of the strategies and their acceptance. This way, the study was able to investigate the specific effects conflict resolution strategy combinations with different robot types and application contexts. The online study format allowed for a text-based strategy presentation without the influence of the real-world implementation into a certain robot prototype (e.g. appearance, specifications, speech synthesis limitations). This meant that the strategy effect could be investigated without biases added by the implementation. When setting up the online studies, standardization of study material was emphasized, by amongst others, ensuring that the robot videos were of the same length, assessing whether the participants got the video displayed correctly, and using validated questionnaires where possible. Manipulation checks regarding robot ratings were successful.

Although the presented studies have provided insights into the acceptance and effectiveness of robot assertiveness, some limitations of the study have to be considered. The extensive testing of fifteen conflict resolution strategies per application context meant that not all participants saw all strategies. This limited the statistical power but, at the same, time diminished potential respondent fatigue. Regarding internal validity, standardization of strategies was difficult with regard to sentence length. Polite speech is naturally more indirect and lengthy as it tends to paraphrase and embellish (Danescu-Niculescu-Mizil et al., 2013). Strategy phrasing has shown to be essential regarding this study’s findings. Thanking dominant (S10.2) was perceived as a negative strategy compared to thanking submissive (S15.2b) which was positively evaluated. Hence, it was reasonable to differentiate between thanking dominant and submissive in Study 2. Consequently, the phrasing for a thankful strategy has to be chosen carefully (present tense vs. subjunctive). For the comparison between the application contexts, it has to be noted that the presented results can only provide first evidence regarding context differences. As the application context was not implemented as an independent variable and the robots differed, further studies are needed which compare both application contexts directly. Although the strategy classification into three valence categories allowed for systematically testing participants’ ratings differed from the expected affective evaluation. Some of the positive strategies were rated more neutrally than expected and none was rated very positively. The categorization based on the human-power asymmetry should not be seen as final but as a working hypothesis that allows for systematically testing. However, it shows the relevance of assessing participant’s perception of strategy valence for future testing of robotic conflict resolution strategies. Finally, as the evaluation was conducted online, external validity might be limited. As only the intention to comply could be measured and videos cannot replace real world encounters, lab and field experiments are needed to replicate results. This holds especially true for physical strategies which might have been difficult to imagine although they were described in relativity to the participants position (e.g. until the robot touches your luggage). Limitations regarding immersion seem likely but that the robot behaviour could trigger reactance and that some strategies (e.g. threat and command) were not even accepted in an online setting with imagined interaction indicates the psychological reality of the participants during the study. It has also been shown in previous HRI studies that imagined interaction with a robot does resemble real HRI with regard to acceptance of the robot, participant’s behaviour toward the robot (Wullenkord and Eyssel, 2019) and negative attitudes towards the robot (Wullenkord et al., 2016).

Therefore, guided imagined interactions seemed to be reasonable for conducting preliminary evaluations of the developed strategies. The intention behind the online format was not to replace real-world testing but to detect strategies that might already be rejected in an imagined situation (which was indeed the case for threat and command) and eliminate them from future research agendas regarding acceptable and effective robotic conflict resolution strategies. Then, for real-world testing, it can be focused on the final best-accepted strategies. Beyond the limitations of online testing, the external validity of the results is questionable as the conflict resolution strategies were examined in a specific situation with specific robots. Therefore, future work might aim to clarify the extent to which results can be generalized to different situations, robots and contexts.

### 6.3 Future Work

Future studies are needed to determine factors that render some robotic conflict resolution strategies more acceptable and effective than others. Hereby, robot, human and situational influences need to be considered. On the robot side, the strategy implementation must be skilfully implemented in terms of speech (e.g. tone of voice), gestures and proximity. Appropriate expression of assertiveness in human conflict resolutions is considered a communication skill that is not trivial to acquire (Pfafman, 2017). For this, it seems reasonable to rely on psychological research not only for strategy development but also for implementation, e.g. training programs to promote appropriate assertiveness at work (Thacker and Yost, 2002; Wilson et al., 2003; Nakamura et al., 2017). Additionally, future work is needed to determine appropriate conflict resolution strategies for more robot types (e.g. androids) and sizes (e.g. miniature, man-sized) which were not represented in the presented studies. Potentially, with an even more varied set of robots than used in the presented studies, robot characteristics like humanness, power of impact and authority might turn out as moderators for strategy effectiveness and acceptance.

On the human side, user personality, robot attitudes and pre-experience, as well as culture, are likely to be of importance for strategy acceptance and effectiveness as they are influential in human negotiations. Here, general personality traits (BIG5, Costa and McCrae, 1985) and specific conflict-related traits such as the conflict type (ROCI-II, Rahim, 1983) have shown to determine individual conflict behaviour (Rahim, 1983; Park and Antonioni, 2007). An integrating style was positively associated with Agreeableness and Extraversion (Park and Antonioni, 2007). Dominating personalities use distributive conflict resolution strategies (Rahim, 1983) and are positively associated with Extraversion but negatively with Agreeableness (Park and Antonioni, 2007). Conceivably, the robot’s strategy has to match the user conflict personality to be effective and accepted. If a dominating negotiator is confronted with an assertive robot, the robot might be less acceptable than if the robot had applied the strategy to a person with an obliging conflict style. In addition, negative attitudes and fears about robots could negatively influence the acceptance of and compliance with assertive robots, since such individuals already tend not to accept non-assertive robots (De Graaf and Allouch, 2013b; Ghazali et al., 2020). Negative attitudes and state anxiety have also shown to negatively influence trust in HRI (Miller et al., 2020). Culture is an additional influence that needs examination in future work. Cultural expectations shape expectations regarding politeness and assertiveness (Lee et al., 2017). Assertiveness must be considered appropriate (e.g. to context and culture), otherwise it can be perceived as aggressive (Pfafman, 2017). An assertive robot might be acceptable in Eurasian countries but could be considered as inappropriate and rude in Asian countries. For Germans and Chinese this has been shown for assertive communication strategies of a small autonomous delivery robot towards pedestrians (Lanzer et al., 2020). Consequently, the presented findings need further confirmation in different samples. Summarizing, future studies are needed to determine the influences of user characteristics on the acceptance of robot assertiveness. Findings could then be used to personalize the robot in the home setting as it has been suggested with other robot characteristics (Ligthart and Truong, 2015).

Situational influences on strategy acceptance and effectiveness are likely to be the conflict scenario (e.g. emergency situations), other application contexts (security robots), repetition and habituation. Apart from the presented scenarios, robot assertiveness could be especially useful for emergency situations. In the public context, for example, security robots might help during an evacuation and might need to be assertive to gain people’s trust and compliance in such a stressful, chaotic situation. In the private context, a service robot might need to be assertive and call an ambulance in case of a medical emergency. To avoid that the results are possibly distorted by the novelty effect of an assertive robot, it is necessary to test whether repeated interaction changes the participants’ attitude and behaviour towards the robot’s assertiveness (e.g. habituation, trust building). If the user benefited from the autonomy and effectiveness of the robot in the past and trust was built up through reliable functioning and appropriate robot actions, the acceptance of the robot’s assertiveness could increase (Ghazali et al., 2020; Kraus et al., 2019; Kraus et al., 2020). Similarly, human-robot power asymmetry might be reduced by habituation when assertive robots become an effective and accepted part of our society. This paper represents the first step towards this goal.

## 7 Conclusion

With future dissemination of service robots in public and private spaces, human-robot goal conflicts will arise. To negotiate acceptable outcomes and for efficient task execution, it might be feasible to apply an assertive robot behaviour under certain circumstances. This study explored different conflict resolution strategies, ranging from polite to assertive, to achieve user compliance and acceptance simultaneously in two application contexts, public and private space. The potential of applying robotic conflict resolution strategies to increase intended compliance with a robot’s request in an acceptable way was shown. Positive and neutral conflict resolution strategies were acceptable and effective in achieving compliance with a robot’s request and should be explored further. Combining strategies based on cognitive mechanisms with politeness seems especially feasible for both application contexts. Only command (S5) and threat (S6) do not seem feasible to be examined further as they were neither effective nor accepted. The perceived interpersonal power of the robot influenced the participants’ decision to comply. Trust and politeness were predictive of strategy acceptance. Concluding, if applied context-sensitively and robot-specifically, robotic conflict resolution strategies as an appropriate expression of robot assertiveness have the potential to solve human-robot goal-conflicts effectively and acceptably. This study represents a first step to designing conflict resolution strategies for future assertive robots. Future work is needed to determine factors that render robot assertiveness acceptable for various users, robots and situations.

## Data Availability

The raw data supporting the conclusions of this article will be made available by the authors, without undue reservation.
